# Cardiovascular Care Disruptions Among U.S. Adults During the COVID-19 Pandemic: Medication Use, Mortality, and Medicare Hospitalization Trends From the Behavioral Risk Factor Surveillance System (BRFSS) Database

**DOI:** 10.7759/cureus.85828

**Published:** 2025-06-12

**Authors:** Efeturi M Okorigba, Chekwube M Obianyo, Said R Tindwa, Padmavathi Mogili, Okelue E Okobi, Anh N Nguyen, Zimakor D Ewuzie, Inemialu M Okhagbuzo, Erhieyovbe Emore

**Affiliations:** 1 Internal Medicine, West Virginia University, Morgantown, USA; 2 Epidemiology and Public Health, Jiann-Ping Hsu College of Public Health, Georgia Southern University, Statesboro, USA; 3 Internal Medicine, Tula State University, Tula, RUS; 4 Internal Medicine, Luminis Health Doctors Community Hospital, Lanham, USA; 5 Family Medicine, IMG Research Academy and Consulting LLC, Homestead, USA; 6 Family Medicine, Larkin Community Hospital Palm Springs Campus, Miami, USA; 7 Family Medicine, Lakeside Medical Center, Belle Glade, USA; 8 Hospital Medicine, Luminis Health, Lanham, USA; 9 Adult Psychiatry, Cygnet Hospital Harrogate, Harrogate, GBR; 10 Internal Medicine, Babcock University, Ogun, NGA; 11 Anatomy, Delta State University, Abraka, NGA

**Keywords:** brfss database, cardiovascular disease, hospitalization, medication use, mortality trends

## Abstract

Background

Cardiovascular diseases (CVDs) are the leading cause of death in the United States, with mortality disproportionately affecting older adults and racial/ethnic minorities. This study analyzes national patterns in CVD medication use, mortality, and hospitalization from 2019 to 2021.

Objectives

To examine patterns of antihypertensive and lipid-lowering medication use, trends in CVD-related mortality (stroke, coronary heart disease, and total heart disease), and hospitalization rates for heart failure among the U.S. population before and during the COVID-19 pandemic.

Methods

This retrospective study utilized Behavioral Risk Factor Surveillance System (BRFSS) data from 2019 to 2021, a nationally representative telephone survey coordinated by the CDC. Trends in cardiovascular medication use were stratified by sex, age, and race/ethnicity. Descriptive statistics, paired t-tests, and one-way ANOVA were used to assess temporal changes. A p-value <0.05 indicates statistical significance. Analyses were conducted using SPSS version 30 (IBM Corp., Armonk, USA).

Results

The use of antihypertensive and cholesterol-lowering medications remained stable overall but declined slightly in some subgroups during 2020. Among adults with high blood pressure, antihypertensive medication use increased from 57.7% (95% CI: 57.1-58.4) in 2019 to 60.4% (95% CI: 59.6-61.1) in 2021. Similarly, cholesterol-lowering medication use rose from 28.9% (95% CI: 28.6-29.2) to 31.0% (95% CI: 30.6-31.3). Heart failure hospitalizations among Medicare beneficiaries aged ≥65 years declined from 27.72 per 1,000 (95% CI: 27.66-27.78) in 2019 to 22.87 (95% CI: 22.81-22.92) in 2020, before increasing to 25.91 (95% CI: 25.85-25.97) in 2021. Cerebrovascular, coronary heart disease, and overall heart disease mortality rates consistently increased from 2019 to 2021, with heart disease deaths rising from 161.5 to 173.8 per 100,000, totaling over 695,000 deaths in 2021.

Conclusions

The COVID-19 pandemic was associated with modest declines in CVD medication use and significant increases in cardiovascular mortality, particularly among high-risk populations. Hospitalizations for heart failure initially decreased but partially recovered by 2021. These findings underscore the necessity for resilient healthcare systems and targeted strategies to mitigate pandemic-related disruptions in chronic cardiovascular care.

## Introduction

Cardiovascular diseases (CVDs) are the leading cause of death globally, accounting for approximately 33% of all deaths in 2019 and causing 20.5 million deaths in 2021, 80% of which occurred in low- and middle-income countries, where infectious diseases nonetheless remain a substantial contributor to morbidity and mortality [1]. In the United States, CVDs remain the primary cause of death, contributing to approximately one in five deaths annually [1]. Specifically, heart disease caused more than 695,000 deaths in 2021, translating to a death every 34 seconds. Stroke, another major contributor to cardiovascular mortality, led to nearly 162,000 deaths in the same year, with an estimated 170,000 silent strokes occurring without noticeable symptoms [2]. The economic burden of CVDs is equally substantial, with the annual cost of healthcare services, medications, and lost productivity exceeding $239 billion in the U.S. alone. Projections indicate that the costs associated with cardiovascular conditions will rise dramatically, with annual healthcare costs potentially quadrupling from $393 billion in 2020 to $1.49 trillion by 2050 [3, 4].

The effective management of cardiovascular conditions is central to reducing mortality and morbidity, which requires timely diagnosis, adherence to evidence-based medication regimens such as antihypertensive and lipid-lowering therapies, and efficient hospital services for acute events like myocardial infarction and heart failure [5]. Despite the availability of clinical guidelines, treatment adherence and healthcare utilization often exhibit significant variations across different demographic, geographic, and socioeconomic groups, leading to disparities in health outcomes [5, 6].

The COVID-19 pandemic has introduced new challenges in managing chronic diseases, including CVD. From early 2020, disruptions in healthcare services, reduced outpatient care, and changes in health-seeking behaviors likely impacted medication adherence, hospitalization patterns, and cardiovascular mortality [7]. Initial reports indicated reductions in emergency visits and elective hospitalizations for cardiovascular conditions, despite rising mortality rates from heart disease. The COVID-19 pandemic significantly disrupted the continuity of care for individuals with chronic diseases, a population for whom consistent medical management is essential. This study aimed to identify key factors that interfered with ongoing care for patients with chronic illnesses during the COVID-19 pandemic.

Two main categories of disruptions were identified: patient-side factors and health system-side factors. Patient-related challenges included negative emotional states such as fear and anxiety, advanced age, disease severity, and the duration of the chronic condition. On the health system side, disruptions included limited access to medical and para-clinical services, shortages in medical supplies, reduced public transportation, and economic hardship leading to decreased care affordability [2]. These disruptions collectively contributed to poorer management of chronic conditions such as hypertension, diabetes, and hyperlipidemia - diseases that are strongly associated with adverse cardiovascular outcomes and increased mortality. However, a comprehensive, population-level understanding of the pandemic’s long-term effects on cardiovascular health is still developing [8].

In this context, the Behavioral Risk Factor Surveillance System (BRFSS), a self-reported, nationally representative survey conducted by the United States Centers for Disease Control and Prevention (CDC), provides a valuable resource to assess trends in CVD medication use. Established in 1984, BRFSS monitors health behaviors, conditions, and preventive services among U.S. adults. With data collected from all 50 states, the District of Columbia, and three U.S. territories, BRFSS is the largest continuous health survey globally, completing over 400,000 adult interviews annually [9, 10].

This study aims to 1) evaluate temporal trends in antihypertensive and lipid-lowering medication use, CVD mortality, and heart failure hospitalizations; and 2) analyze associations between demographic factors (age, sex, race/ethnicity) and these trends. These aims guide this study to investigate trends in CVD medication use, mortality, and hospitalization rates among the U.S. population, with a special focus on Medicare beneficiaries aged 65 and older from 2019 to 2021. The analysis focuses on changes in self-reported use of antihypertensive and cholesterol-lowering medications, age-adjusted mortality trends from stroke, coronary heart disease (CHD), and total heart disease, and hospitalization rates for heart failure among Medicare beneficiaries aged 65 and older. Findings will inform public health strategies and targeted interventions to strengthen cardiovascular care resilience during and beyond pandemic disruptions.

## Materials and methods

Study design and database

This retrospective study employed data from the BRFSS, a nationally representative, state-based telephone survey coordinated by the CDC. Data are self-reported, and when a selected respondent was unable to answer, a proxy (next of kin or another knowledgeable household member) provided telephonic responses. Verbal informed consent was obtained from all respondents or proxies before survey administration. The BRFSS collects annual data on health-related behaviors, chronic health conditions, and preventive service usage among the non-institutionalized U.S. population. For this analysis, we utilized BRFSS datasets from two survey years (2019 and 2021) to examine trends in CVD medication use, alongside national mortality and hospitalization patterns among U.S. adults, with a focus on older adults [10].

Study population and variables 

The study population comprised adults aged ≥18 years who completed the BRFSS in 2019 and 2021. Key variables included self-reported diagnoses of hypertension and high cholesterol and current use of antihypertensive or cholesterol-lowering medications. Data were stratified by sex, age group, and race/ethnicity to assess demographic disparities. The primary outcomes were the proportions of adults with high blood pressure or high cholesterol who reported medication usage. Secondary outcomes included age-adjusted mortality trends and heart failure hospitalization rates. Hypertension and high cholesterol diagnoses were defined by self-report of a provider diagnosis. Medication-use variables were coded ‘yes’ if respondents answered affirmatively to current use. Missing values (<5% per variable) were handled via listwise deletion, given the survey’s complex weighting to maintain national representativeness.

Inclusion and exclusion criteria

Participants were included if they were adults (≥18 years) who responded in 2019 or 2021 (exit point) and completed questions on hypertension, high cholesterol, and medication use. Individuals were included if they reported a diagnosis of either condition and provided valid responses about medication use. We did not include 2020 BRFSS data due to pandemic-related survey disruptions and incompleteness. Participants were excluded if key variables were missing or if responses were incomplete, inconsistent, or indicated "don't know/not sure" or "refused" for relevant questions. Institutionalized individuals and those under 18 were excluded, consistent with BRFSS methodology, which focuses on the non-institutionalized U.S. population. Of 820,000 total respondents in 2019 and 2021 combined, 37,500 (4.6%) were excluded for missing key variables. Mortality and hospitalization analyses used completely separate CDC and Medicare administrative datasets, analyzed in parallel, with triangulation at the population level rather than individual linkage.

BRFSS questionnaire structure

The BRFSS questionnaire consists of three parts: (1) a core component, which includes standardized annual and rotating questions used by all states; (2) optional modules, covering specific topics like diabetes and cancer, which states may choose to include; and (3) state-added questions, created independently by states and not reviewed or reported by the CDC.

Mortality and hospitalization data

Mortality data focused on age-adjusted death rates for stroke, coronary heart disease, and total heart disease per 1000 population. Data were categorized by year (2019-2021), sex, age group (0-44, 45-64, ≥65 years), and race/ethnicity. Hospitalization data were analyzed for Medicare beneficiaries aged 65 years and older because Medicare claims allow reliable capture of hospitalizations in this group. Hospitalization rates for heart failure, based on principal diagnosis codes, were reported per 1,000 beneficiaries and stratified by sex and race/ethnicity to identify disparities and temporal patterns.

Statistical analysis

Descriptive statistics, including frequencies and proportions, were used to summarize participant characteristics. Weighted prevalence estimates and 95% confidence intervals (CIs) were calculated to ensure national representation. Temporal trends were assessed using paired t-tests and one-way ANOVA for group comparisons. A p-value <0.05 was considered statistically significant. All analyses were conducted using SPSS version 30 (IBM Corp., Armonk, USA).

Ethical consideration

This study involved human subjects and was conducted in accordance with the principles outlined in the Declaration of Helsinki. As this study used de-identified, publicly available data, it was exempt from institutional review board (IRB) approval. Although the study was retrospective in nature and utilized de-identified data, informed consent was obtained where applicable. All data were handled with strict confidentiality to ensure the privacy and anonymity of the participants.

## Results

Trends in cardiovascular medication use among U.S. adults

Analysis of the BRFSS data revealed an upward trend in cardiovascular disease medication use between 2019 and 2021. Among adults with high blood pressure, the proportion taking antihypertensive medication increased from 57.7% (95% CI: 57.1-58.4) in 2019 to 60.4% (95% CI: 59.6-61.1) in 2021. Similarly, those taking medication for high cholesterol rose from 28.9% (95% CI: 28.6-29.2) to 31.0% (95% CI: 30.6-31.3). Women reported higher antihypertensive medication use than men in both years, with a statistically significant difference (P=0.047). However, men consistently showed higher use of cholesterol-lowering medications. 

Racial disparities were also observed. Black non-Hispanic individuals had the highest antihypertensive medication use (64.3% in 2019, 67.5% in 2021), while Hispanic adults showed minimal change. Statin use increased notably among Hispanic adults, from 29.3% to 32.2%, though the change was not statistically significant (P=0.437).

Age was a strong determinant of medication use. Adults aged ≥65 years had the highest prevalence of both antihypertensive (92.5%) and cholesterol medication use (53.1%) by 2021. These age-related trends were statistically significant (P<0.001). Table 1 indicates the trends in cardiovascular medication use among US adults by demographic characteristics. 

**Table 1:** Trends in Cardiovascular Medication Use Among U.S. Adults by Demographic Characteristics, BRFSS 2019 vs. 2021 CI: Confidence interval, *: A p-value <0.05 indicates statistical significance

Category	Parameters	Taking medicine to control high blood pressure among adults with high blood pressure		Taking medicine for high cholesterol among adults	
	Category	2019 (% (95% CI))	2021 (% (95% CI))	2019 (% (95% CI))	2021 (% (95% CI))
Total	Overall	57.7 (57.1-58.4)	60.4 (59.6-61.1)	28.9 (28.6-29.2)	31.0 (30.6-31.3)
Sex	Male	55.0 (54.2-55.8)	57.3 (56.4-58.1)	30.5 (30.0-30.9)	32.4 (31.9-32.8)
	Female	61.8 (60.7-62.9)	65.2 (63.9-66.4)	27.4 (27.0-27.8)	29.6 (29.2-30.1)
	P-value	P=0.047^*^		P=0.047^*^	
	Statistical test value	t stat=-13.36		t stat=-13.36	
Race/Ethnicity	White, non-Hispanic	56.1 (55.4-56.8)	59.6 (58.8-60.4)	28.9 (28.5-29.2)	30.8 (30.4-31.2)
	Black, non-Hispanic	64.3 (62.4-66.1)	67.5 (65.4-69.5)	27.8 (26.9-28.7)	29.2 (28.3-30.1)
	Hispanic	56.8 (55.0-58.6)	56.4 (54.6-58.3)	29.3 (28.4-30.3)	32.2 (31.2-33.2)
	P-value	P=0.031^*^		P=0.437	
	Statistical test value	F=13.49		F=1.10	
Age Group	18-44 years	38.1 (36.8-39.3)	42.4 (41.0-43.8)	15.9 (15.5-16.3)	18.4 (17.9-18.9)
	45-64 years	80.0 (79.3-80.7)	82.0 (81.4-82.7)	39.8 (39.3-40.3)	41.3 (40.8-41.9)
	≥65 years	92.1 (91.7-92.5)	92.5 (92.1-92.9)	50.6 (50.1-51.1)	53.1 (52.5-53.8)
	P-value	P=0.0004^*^		P=0.0002^*^	
	Statistical test value	F=397.12		F=254.8	

Heart failure hospitalization rates among Medicare beneficiaries

Hospitalization rates for heart failure as the principal diagnosis among Medicare beneficiaries aged 65 years and older declined sharply in 2020 during the COVID-19 pandemic. In 2019, there were 805,708 cases, corresponding to a rate of 27.72 cases per 1,000 population (95% CI: 27.66-27.78). This number declined in 2020 to 661,335 cases, with a lower rate of 22.87 per 1,000 (95% CI: 22.81-22.92). In 2021, the number of cases rose again to 725,404, with a corresponding rate of 25.91 per 1,000 (95% CI: 25.85-25.97). These figures indicate a sharp decline during 2020, followed by a partial recovery in 2021.

Sex-based differences were statistically significant (P=0.012). Males consistently had higher hospitalization rates than females across all years. In 2021, the rate was 27.39 per 1,000 among males versus 24.67 among females.

Racial and ethnic disparities were pronounced. Black non-Hispanic beneficiaries had the highest hospitalization rates throughout the period, peaking at 55.85 per 1,000 in 2021. Hispanic beneficiaries followed, with a rate of 33.68 in 2021. White non-Hispanic beneficiaries consistently had lower rates (23.83 in 2021), while Asian or Pacific Islander groups had the lowest (18.40 in 2021). All racial/ethnic differences were statistically significant (P<0.05, F=68.72). These findings highlight a temporary decline in heart failure hospitalizations during the pandemic and a subsequent rebound, with persistent disparities by sex and race/ethnicity among older adults. Table 2 indicates the heart failure hospitalization rates among Medicare beneficiaries aged 65 years and older by demographics, 2019-2021.

**Table 2:** Heart Failure Hospitalization Rates Among Medicare Beneficiaries Aged ≥65 Years by Demographics, 2019-2021 CI: Confidence interval, *: A p-value <0.05 indicates statistical significance

Category		2019 (cases per 1,000 (95% CI))	2020 (cases per 1,000 (95% CI))	2021 (cases per 1,000 (95% CI))
Total	Overall	27.72 (27.66-27.78)	22.87 (22.81-22.92)	25.91 (25.85-25.97)
	Number	805,708	661,335	725,404
Sex	Male	29.92 (29.83-30.02)	24.57 (24.48-24.65)	27.39 (27.30-27.48)
	Female	25.95 (25.88-26.03)	21.47 (21.40-21.54)	24.67 (24.59-24.75)
	P-value	P=0.012^*^		
	Statistical test value	t stat=8.82		
Race/Ethnicity	White, non-Hispanic	25.62 (25.56-25.68)	21.05 (20.99-21.10)	23.83 (23.77-23.89)
	Black, non-Hispanic	55.46 (55.15-55.76)	48.18 (47.88-48.47)	55.85 (55.52-56.18)
	Hispanic	36.31 (35.79-36.84)	29.91 (29.43-30.40)	33.68 (33.16-34.20)
	American Indian or Alaska Native, non-Hispanic	26.46 (25.62-27.30)	23.93 (23.13-24.74)	24.28 (23.45-25.12)
	Asian or Pacific Islander, non-Hispanic	20.04 (19.70-20.39)	15.54 (15.24-15.85)	18.40 (18.06-18.73)
	P-value	P<0.05^*^		
	Statistical test value	F=68.72		

Trends in mortality rates for stroke, coronary heart disease

Between 2019 and 2021, cerebrovascular disease (stroke), CHD, and overall heart disease mortality rates among the U.S. population showed a consistent upward trend (Table 3). In 2019, cerebrovascular disease (stroke) accounted for 150,005 deaths, corresponding to a rate of 37.0 cases per 100,000 population (95% CI: 36.8-37.1). This increased to 160,264 deaths (38.8 per 100,000; 95% CI: 38.6-39.0) in 2020 and further to 162,890 deaths (41.1 per 100,000; 95% CI: 40.9-41.3) in 2021. Coronary heart disease mortality followed a similar trajectory, with 360,900 deaths in 2019 (88.0 per 100,000; 95% CI: 87.7-88.3), rising to 382,820 (91.8 per 100,000; 95% CI: 91.5-92.1) in 2020 and 375,476 (92.8 per 100,000; 95% CI: 92.5-93.1) in 2021. Deaths due to all diseases of the heart were highest overall, totaling 659,041 in 2019 (161.5 per 100,000; 95% CI: 161.1-161.9), increasing to 696,962 in 2020 (168.2 per 100,000; 95% CI: 167.8-168.6), and reaching 695,547 in 2021 (173.8 per 100,000; 95% CI: 173.4-174.2). These trends suggest a consistent rise in cardiovascular mortality, particularly during the COVID-19 pandemic period.

Sex-based differences were significant across all years. Males had higher mortality rates than females in all three categories. In 2021, male stroke mortality was 41.5 versus 40.2 per 100,000 in females (P=0.028), CHD mortality was 127.4 vs. 64.7 (P=0.0003), and total heart disease mortality was 219.5 vs. 135.6 per 100,000 (P=0.0004).

Age was a dominant risk factor. Among adults ≥65 years, stroke mortality reached 249.4 per 100,000 in 2021, CHD mortality was 532.2, and overall heart disease mortality was 990.6. Comparatively, participants aged 0-44 had dramatically lower rates (1.7, 3.4, and 9.6 per 100,000, respectively). All age-related differences were highly significant (P<0.05).

Racial and ethnic disparities persisted. Black non-Hispanic adults had the highest stroke mortality at 59.6 per 100,000 in 2021, significantly exceeding the rate in White non-Hispanics (39.8) and Hispanics (36.1). CHD mortality for Black non-Hispanics rose to 110.5 in 2021, while total heart disease mortality was 226.2, substantially higher than in Asians (85.5) and Hispanics (119.0). Native Hawaiian/Pacific Islanders and American Indian/Alaska Native groups also experienced high overall heart disease mortality (182.5 and 155.2, respectively, in 2021). Overall, the upward trends in cardiovascular mortality emphasize the continued burden of heart and cerebrovascular diseases in the U.S., with notable disparities by sex, age, and race/ethnicity. Table 3 indicates the trends in mortality rates for stroke, coronary heart disease, and total heart disease.

**Table 3:** Trends in mortality rates for stroke, coronary heart disease, and total heart disease among U.S. adults CI: Confidence interval, *: A p-value <0.05 indicates statistical significance

Category	Parameters	Cerebrovascular disease (stroke) mortality among all people, underlying cause			Coronary heart disease mortality among all people, underlying cause			Diseases of the heart mortality among all people, underlying cause		
	Variables	2019 (cases per 100,000 (95% CI))	2020 (cases per 100,000 (95% CI))	2021 (cases per 100,000 (95% CI))	2019 (cases per 100,000 (95% CI))	2020 (cases per 100,000 (95% CI))	2021 (cases per 100,000 (95% CI))	2019 (cases per 100,000 (95% CI))	2020 (cases per 100,000 (95% CI))	2021 (cases per 100,000 (95% CI))
Total	Overall	37 (36.8-37.1)	38.8 (38.6-39.0)	41.1 (40.9-41.3)	88 (87.7-88.3)	91.8 (91.5-92.1)	92.8 (92.5-93.1)	161.5 (161.1-161.9)	168.2 (167.8-168.6)	173.8 (173.4-174.2)
	Number	150,005	160,264	162,890	360,900	382,820	375,476	659,041	696,962	695,547
Sex	Male	37.6 (37.3-37.9)	39.8 (39.5-40.1)	41.5 (41.2-41.8)	120.9 (120.3-121.4)	126.2 (125.7-126.7)	127.4 (126.8-127.9)	204.8 (204.1-205.5)	214.2 (213.5-214.9)	219.5 (218.8-220.3)
	Female	35.8 (35.6-36.1)	37.4 (37.2-37.7)	40.2 (39.9-40.5)	61.7 (61.4-62.0)	64.1 (63.7-64.4)	64.7 (64.4-65.1)	126.2 (125.8-126.7)	130.2 (129.8-130.7)	135.6 (135.2-136.1)
	P-value	P=0.028^*^			P=0.0003^*^			P=0.0004^*^		
	Statistical test value	t stat=5.76			t stat=56.75			t stat=46.04		
Age Group	0-44 years	1.4 (1.4-1.5)	1.6 (1.5-1.6)	1.7 (1.6-1.7)	2.9 (2.8-3.0)	3.3 (3.2-3.3)	3.4 (3.3-3.5)	8.1 (8.0-8.2)	9.2 (9.1-9.3)	9.6 (9.4-9.7)
	45-64 years	21.7 (21.4-22.0)	24.0 (23.6-24.3)	24.4 (24.1-24.8)	79.1 (78.5-79.7)	86.7 (86.1-87.4)	85.9 (85.3-86.5)	134.4 (133.6-135.2)	148.3 (147.4-149.1)	148.4 (147.5-149.2)
	≥65 years	239 (237.7-240.3)	246.8 (245.5-248.1)	249.4 (248.0-250.7)	535.4 (533.4-537.3)	547.6 (545.6-549.5)	532.2 (530.3-534.1)	983.4 (980.7-986.0)	1000.1 (997.5-1002.8)	990.6 (988.0-993.2)
	P-value	P<0.05^*^			P<0.05^*^			P<0.05^*^		
	Statistical test value	F=3288			F=8968			F=18820		
Race/Ethnicity	White, non-Hispanic	35.7 (35.5-35.9)	37.2 (37.0-37.4)	39.8 (39.6-40.1)	91.0 (90.7-91.3)	93.4 (93.0-93.7)	96.2 (95.8-96.6)	166.4 (166.0-166.9)	170.8 (170.3-171.3)	179.8 (179.3-180.3)
	Black, non-Hispanic	53.1 (52.4-53.9)	57.6 (56.8-58.3)	59.6 (58.8-60.4)	103.9 (102.8-104.9)	115.8 (114.7-116.9)	110.5 (109.5-111.6)	208.6 (207.1-210.1)	231.6 (230.1-233.2)	226.2 (224.6-227.7)
	Hispanic	32.8 (32.2-33.4)	34.9 (34.3-35.5)	36.1 (35.4-36.7)	67.8 (66.9-68.6)	75.6 (74.7-76.5)	70.6 (69.7-71.5)	111.3 (110.2-112.4)	122.7 (121.6-123.9)	119.0 (117.8-120.1)
	Asian, non-Hispanic	29.3 (28.5-30.1)	31.6 (30.8-32.3)	32.6 (31.8-33.4)	49.1 (48.1-50.1)	54.8 (53.8-55.9)	52.7 (51.7-53.7)	79.2 (77.9-80.5)	86.9 (85.5-88.2)	85.5 (84.2-86.8)
	Hawaiian or Pacific Islander, non-Hispanic	50.9 (44.3-57.6)	47.0 (40.9-53.2)	46.7 (40.7-52.8)	97.4 (88.6-106.2)	97.0 (88.4-105.6)	98.8 (90.2-107.4)	168.5 (156.9-180.2)	173.5 (162.0-185.1)	182.5 (170.8-194.2)
	American Indian or Alaska Native, non-Hispanic	31.4 (29.0-33.8)	34.0 (31.5-36.4)	34.6 (32.1-37.1)	79.4 (75.6-83.2)	82.6 (78.9-86.4)	81.7 (78.0-85.4)	141.6 (136.5-146.6)	148.4 (143.3-153.4)	155.2 (150.1-160.4)
	Multiracial, non-Hispanic	17.8 (16.3-19.3)	18.9 (17.4-20.4)	20.3 (18.8-21.8)	38.3 (36.2-40.5)	42.1 (39.9-44.3)	40.7 (38.5-42.8)	69.4 (66.6-72.3)	76.9 (73.9-79.8)	74.9 (72.0-77.7)
	P-value	P<0.05^*^			P<0.05^*^			P<0.05^*^		
	Statistical test value	F=75.6			F=180			F=169.17		

## Discussion

This study provides an integrated overview of trends in CVD medication use, mortality, and hospitalization among the U.S. population, with a specific focus on the period during which data were available amidst the impact of the COVID-19 pandemic. The findings reveal significant disruptions in CVD care patterns and outcomes, many of which align closely with the onset and progression of the pandemic. Notably, the pandemic exacerbated existing health disparities and had a considerable impact on CVD outcomes, particularly in certain demographic groups [7-10 ].

A key finding of this study was the increase in the use of medications for hypertension and hyperlipidemia during the pandemic period. Thus, from 2019 to 2021, the proportion of adults reporting blood pressure and cholesterol-lowering medications showed a modest but consistent upward trend. This pattern suggests that many individuals maintained or even improved adherence to prescribed cardiovascular therapies despite the widespread disruptions to healthcare delivery during the COVID-19 pandemic. Additionally, this finding may reflect increased health awareness, expanded telemedicine services, or policy adjustments that facilitated prescription refills and medication access during public health emergencies [11, 12]. Furthermore, the observed gender differences, higher antihypertensive use among women versus higher cholesterol‑lowering use among men, may reflect disparities in treatment‑seeking behavior, prescribing practices, or risk perception. Understanding these sex‑specific patterns will be essential to tailor outreach and reduce inequities in cardiovascular prevention.

The COVID-19 pandemic compelled many healthcare providers to rapidly adopt telemedicine, including telephone and video consultations, to maintain continuity of care. This urgent shift also created an opportunity to address longstanding shortcomings in traditional office-based care, particularly the poor management of chronic diseases [8, 12]. For example, only about 50% of patients with chronic conditions adhere to their prescribed medications [9-12]. Among individuals with hypertension, fewer than half achieve their blood pressure goals, and medication non-adherence contributes to over $100 billion in annual healthcare costs [9-12]. Even highly evidence-based treatments, such as statin therapy, show only a 50% adherence rate among patients with established cardiovascular disease [9-11]. Hypertension, as a chronic condition, stands to benefit significantly from frequent telehealth visits.

Research has demonstrated that self-monitoring not only improves blood pressure (BP) control but also enhances medication adherence [8, 9]. When self-monitoring is combined with telemonitoring, patients who regularly measure their BP at home exhibit statistically significant improvements in hypertension management [10]. Notably, the greatest reductions in blood pressure are observed in patients who engage in home monitoring alongside regular co-interventions, rather than in those who only self-monitor without additional support [13]. Continuity of care (COC) plays a vital role in chronic disease outcomes. Research has demonstrated that higher COC is associated with reduced risks of cardiovascular disease (CVD), coronary heart disease (CHD), and stroke in patients newly diagnosed with hypertension. It is also linked to improved adherence to antihypertensive medications, underscoring the need to prioritize COC to reduce cardiovascular complications [14].

Despite its benefits, telemedicine cannot fully replace in-person visits. Physical examinations remain essential for many clinical evaluations, and face-to-face interactions carry emotional and psychological value that telemedicine cannot replicate. To strengthen telehealth's role, health systems will need to adopt home-based blood draws, remote imaging, and patient-reported outcomes. Moreover, patient satisfaction and perceived adequacy of telehealth should guide its integration, as many patients are well-positioned to judge whether a remote consultation meets their needs [13].

Although telemedicine use surged during the pandemic and has become more established globally, several barriers remain. These include inequitable access to digital technologies, insufficient digital literacy among users, inadequate reimbursement policies, privacy and cybersecurity concerns, and the need for better integration with existing healthcare infrastructure [15].

Furthermore, mortality data revealed an alarming increase in age-adjusted death rates across major cardiovascular categories between 2019 and 2021. This reversal of previous declines in mortality rates is indicative of the indirect toll the pandemic took on cardiovascular health. Delays in emergency care, overwhelmed healthcare systems, and COVID-19-related cardiac complications, particularly among vulnerable populations, likely contributed to these increased mortality rates [16]. National data indicate roughly 90,000 “excess” cardiovascular deaths (approximately 5% above the expected baseline) occurred from March 2020 to March 2022 [17]. Notably, males consistently exhibited higher mortality than females, especially for coronary heart disease, suggesting possible differences in underlying risk profiles, healthcare access, or treatment adherence. Moreover, adults aged ≥65 experienced mortality rates over 100‑fold greater than those aged 0-44, underscoring the urgent need for age‑targeted prevention and management strategies in both clinical and public‑health settings. This surge encompassed significant increases in both heart disease and stroke mortality rates, and pandemic-related disruption in care appears to be the major driver [18].

Patients with symptoms were unable to present to the hospital due to fear of this virus or the lockdown, leading to missed chances for early intervention [18]. Even among those who did present, hospitals were frequently strained, and timely reperfusion therapy (e.g., primary percutaneous coronary intervention (PCI) for ST-elevation myocardial infarction (STEMI) or thrombolysis/thrombectomy for stroke) was at times delayed or deferred [18]. During the peak of the pandemic, studies showed a marked drop in treated STEMI cases (≈19% fewer primary PCI procedures), implying that more infarctions went untreated or undertreated [19]. Such delays in reperfusion inevitably translated into larger infarct sizes, greater post-event complications, and higher acute mortality. In addition to these indirect effects, COVID-19 infection has been found to directly injure the cardiovascular system. COVID-19 is now recognized to precipitate myocarditis, arrhythmias, and endothelial injury with a prothrombotic state [20]. This is associated with increased risk of arrhythmic sudden cardiac death, heart failure, and ischemic events (STEMI). All these contributed to the excess in cardiovascular deaths during this period. This trend is corroborated by Kobo et al., who reported a 5.9% increase in cardiovascular mortality during the pandemic, with substantial increases in mortality from hypertension and cerebrovascular diseases, particularly in males and younger populations in the U.S. [21].

The United States was not alone in recording an increase in cardiovascular mortality during this period. It varied by region. For instance, a study by Furuse showed that excess cardiovascular deaths in 2020 ranged from as low as ~0.7% above baseline in some high-income countries to over 20% in hard-hit regions [22]. These findings underscore the compounded risks faced by individuals with pre-existing cardiovascular conditions during the pandemic [21, 23].

Race- and ethnicity-stratified analyses further revealed persistent disparities in cardiovascular outcomes. Black, non-Hispanic individuals experienced significantly higher mortality rates compared to White individuals across all CVD categories. For instance, in 2021, stroke-related mortality was 59.6 per 100,000 for Black individuals, compared to 39.8 per 100,000 for White individuals. Similarly, heart disease mortality was 226.2 per 100,000 among Black individuals versus 179.8 per 100,000 in White adults. These findings are consistent with previous studies highlighting the elevated cardiovascular risk and mortality rates among Black populations [24]. Additionally, disparities in CVD outcomes were also observed in Hispanic and American Indian/Alaska Native populations, echoing the findings of Urhoghide et al. (2021), who reported regional and racial variations in cardiovascular mortality, with particularly high mortality rates among African Americans with hypertension [24].

These disparities in cardiovascular outcomes among Blacks, Hispanics, and other people of color have been linked to social and structural barriers that limit access to economic stability, education, healthcare, safe physical environments, and social support [25, 26]. These, collectively referred to as the social determinant of health (SDOH), are defined as the conditions in the environments where people are born, live, learn, work, play, worship, and age that affect a wide range of health, functioning, and quality of life outcomes and risks by both the Centers for Disease Control and Prevention and the World Health Organization [27]. Blacks, Hispanics, and people of color disproportionately lack access to health care, safe living and physical environments, quality education, and equal employment opportunities, and are predisposed to poor health outcomes, including cardiovascular disease (CVD).

Socioeconomic status (SES), including social mobility, was noted to have one of the strongest impacts in shaping cardiovascular health because it influences access to care, safe housing, and other stressors that increase cardiovascular disease risks [27]. Although more than 50 years have passed since work was initiated to address the identified health disparities, Blacks, Hispanics, and other minority groups continue to face greater social disadvantages that impact the development, progression, and mortality of CVD [27]. Javed et al. attributed this persistence to overlooking the social determinants of health (SDOH) in clinical care and ignoring the impact of structural racism in policy-making and practice [27]. And concluding that without properly addressing the reality of structural racism in policy-making and practice, achieving genuine health equity will remain elusive.

The patterns of hospitalization for heart failure also reflected the broader trends in mortality [24, 28]. Data from Medicare beneficiaries showed a significant decline in heart failure hospitalizations in 2020, from 27.72 per 1,000 beneficiaries to 22.87 per 1,000. This reduction likely resulted from a combination of factors, including the widespread avoidance of hospital settings due to fear of COVID-19 infection and the reduction of elective procedures during the early pandemic phase. However, hospitalization rates rebounded in 2021, suggesting that the delayed care during 2020 contributed to worsening conditions that ultimately required acute intervention. Racial disparities persisted in hospitalization rates, with Black and Hispanic patients experiencing consistently higher hospitalization rates compared to their White counterparts. These disparities further emphasize the need for targeted interventions to improve access to healthcare for minority populations [29].

The findings of this study are consistent with previous literature, which has highlighted the negative impact of the COVID-19 pandemic on healthcare utilization and cardiovascular outcomes. Czeisler et al. reported that a significant proportion of U.S. adults delayed or avoided medical care during the early stages of the pandemic, which likely contributed to excess non-COVID mortality, including deaths from cardiovascular diseases [29]. Similarly, research by Hennecken et al. indicated increased mortality from ischemic heart disease and hypertensive disease in underserved populations during 2020 [30]. These trends are echoed in our study, which links the delayed hospitalizations to increased cardiovascular mortality during the pandemic [30, 31]. The American Heart Association’s 2022 update also documented the stagnation in cardiovascular health progress during the pandemic years, emphasizing the reversal of previously steady declines in CVD mortality [32]. This raises significant concerns about the long-term public health implications, particularly as the U.S. population continues to age and the burden of chronic diseases like hypertension and heart disease grows [32].

Future directions

We propose enhancing telehealth impact by improving digital literacy via community training; securing lasting reimbursement parity and incentives for remote monitoring; integrating home‑based diagnostics and wearable data into health records; strengthening linkage of survey, claims, and vital‐stats data for real‑time surveillance; and launching culturally tailored outreach and community‑health‑worker initiatives to close equity gaps in medication adherence and follow‑up care.

Strengths and limitations of the study

Despite the strengths of this study, including the use of nationally representative datasets and the triangulation of self-reported medication use, mortality, and hospitalization data, several limitations warrant consideration. First, BRFSS relies on self-reported data, which may be subject to recall bias and inaccuracies. Second, the cross-sectional nature of the datasets limits the ability to establish direct causal relationships between medication adherence and mortality or hospitalization outcomes. Third, the lack of individual-level data linkage between BRFSS and administrative datasets means that this analysis focuses on population-level trends rather than individual trajectories. Medication‑use data are self‑reported and may be subject to recall or social‑desirability bias, potentially overestimating true adherence.

Nonetheless, the convergence of evidence from multiple high-quality sources reinforces the validity of our conclusions. When considered together, these findings highlight the profound disruptions caused by the COVID-19 pandemic on cardiovascular care, including declines in medication adherence, delays in care, and increased mortality. These disruptions disproportionately affected vulnerable populations, emphasizing the need for more robust healthcare systems and targeted interventions to reduce health disparities, particularly in cardiovascular health, moving forward.

## Conclusions

This study highlights the adverse impact of the COVID-19 pandemic on cardiovascular disease management and outcomes in the U.S., with significant increases in mortality, temporary declines in hospitalizations, and modest reductions in medication use. The disproportionate burden borne by racial and ethnic minorities and older adults underscores the urgent need to reinforce cardiovascular preventive care and equitable access to services. Recovery efforts must prioritize rebuilding chronic disease infrastructure, enhancing outreach in underserved communities, and investing in policies that address the root causes of health inequities.
